# Adverse events associated with JAK inhibitors in 126,815 reports from the WHO pharmacovigilance database

**DOI:** 10.1038/s41598-022-10777-w

**Published:** 2022-05-03

**Authors:** Léa Hoisnard, Bénédicte Lebrun-Vignes, Sébastien Maury, Matthieu Mahevas, Khalil El Karoui, Lydia Roy, Anissa Zarour, Marc Michel, José L. Cohen, Aurélien Amiot, Pascal Claudepierre, Pierre Wolkenstein, Philippe Grimbert, Emilie Sbidian

**Affiliations:** 1 Fédération Hospitalo-Universitaire TRUE InnovaTive theRapy for immUne disordErs, Assistance Publique-Hôpitaux de Paris (AP-HP), Henri Mondor Hospital, 94010 Créteil, France; 2 INSERM, Centre d’Investigation Clinique 1430, 94010 Créteil, France; 3 EpiDermE Epidemiology in Dermatology and Evaluation of Therapeutics, EA7379, Paris Est Créteil University UPEC, 94010 Créteil, France; 4 Department of Pharmacology, Pharmacovigilance Unit, Assistance Publique-Hôpitaux de Paris (AP-HP), Pitié-Salpêtrière Hospital, Paris, France; 5 Hematology Department, Assistance Publique-Hôpitaux de Paris (AP-HP), Henri Mondor Hospital & Faculté de Santé, UPEC (Université Paris Est Créteil), 94010 Créteil, France; 6 Department of Internal Medicine, Assistance Publique-Hôpitaux de Paris (AP-HP), Henri Mondor Hospital, 94010 Créteil, France; 7 Department of Nephrology and Renal Transplantation, Assistance Publique-Hôpitaux de Paris (AP-HP), Henri Mondor Hospital, 94010 Créteil, France; 8 UPEC (Université Paris Est Créteil), UMR-S955, 94010 Créteil, France; 9 INSERM (Institut National de la Santé et de la Recherche Médicale) U955, Institut Mondor de Recherche Biomédicale (IMRB), 94010 Créteil, France; 10 INSERM, Centre d’Investigation Clinique Biothérapie 1430, 94010 Créteil, France; 11 Department of Gastroenterology, Henri Mondor Hospital, Assistance Publique-Hôpitaux de Paris (AP-HP), EA7375 and Université Paris Est, Creteil, France; 12 Department of Rheumatology, Henri Mondor Hospital, Assistance Publique-Hôpitaux de Paris (AP-HP), 94000 Créteil, France; 13 Department of Dermatology, Assistance Publique-Hôpitaux de Paris (AP-HP), Henri Mondor Hospital, 94010 Créteil, France

**Keywords:** Epidemiology, Adverse effects, Biological therapy, Immunological disorders

## Abstract

Increasing number of Janus kinase (JAK) inhibitors have been approved for chronic haematopoietic neoplasms and inflammatory/autoimmune diseases. We aimed to assess safety of the first three approved JAK inhibitors: ruxolitinib, tofacitinib and baricitinib. In this retrospective observational study, pharmacovigilance data were extracted from the World Health Organization database. Adverse events are classified according to Medical Dictionary for Regulatory Activities hierarchy. Until February 28, 2021, all Individual Case Safety Reports [ICSRs] with the suspected drug ruxolitinib, tofacitinib or baricitinib were included. Disproportionality analysis was performed and the information component (IC) was estimated. Adverse events were considered a significant signal if the lower end of the 95% credibility interval of the IC (IC025) was positive. We identified 126,815 ICSRs involving JAK inhibitors. Ruxolitinib, tofacitinib and baricitinib were associated with infectious adverse events (IC025 1.7, especially with viral [herpes and influenza], fungal, and mycobacterial infectious disorders); musculoskeletal and connective tissue disorders (IC025 1.1); embolism and thrombosis (IC025 0.4); and neoplasms (IC025 0.8, especially malignant skin neoplasms). Tofacitinib was associated with gastrointestinal perforation events (IC025 1.5). We did not find a significant increase in the reporting of major cardiovascular events. We identified significant association between adverse events and ruxolitinib, tofacinitib and baricitinib in international pharmacovigilance database.

## Introduction

For a decade, growing interest in clinical immunology and rheumatology regarding targeted therapies to block cytokines and their signaling have led to the development and use of Janus kinase (JAK) inhibitors. Janus kinases are cytokine transmembrane receptors: JAK1, JAK2, JAK3 and TYK2. JAK-STAT (signal transducer and activator of transcription) pathway plays roles in orchestrating of immune system, cell proliferation and haematopoiesis^1^. JAK-STAT pathway is implicated in the pathogenesis of inflammatory and autoimmune diseases including rheumatoid arthritis, psoriasis, and inflammatory bowel disease as well as malignancies^1^.

Three of the JAK inhibitors have been approved for a few years by the US Food and Drug Administration/European Medicines Agency (FDA/EMA). Tofacitinib, a selective JAK1 and JAK 3 inhibitor, has been approved for treating rheumatoid arthritis, psoriatic arthritis, and ulcerative colitis. Ruxolitinib, a selective JAK1 and JAK2 inhibitor, has been approved for treating myelofibrosis and polycythemia vera. Baricitinib selectively inhibits JAK 1 and JAK 2 and has been approved for treating rheumatoid arthritis and atopic dermatitis. The success of JAK inhibitors in the treatment of inflammatory diseases or malignancies demonstrates that intracellular signaling pathways can be targeted to treat inflammatory and autoimmune diseases. Perspectives for the use of these three JAK inhibitors are now wider, for other inflammatory/autoimmune diseases^2,3^. Moreover, increasing number of JAK inhibitors have been recently approved or assessed in clinical trials, including research into cancer treatment^4–7^. In this context of the intensive development of these JAK inhibitors, safety data are crucial.

The first three approved JAK inhibitors (ruxolitinib anti-JAK1,2, tofacitinib anti-JAK1,3, and baricitinib anti-JAK1,2,) can offer sufficient perspectives for safety studies, for patients who participated into clinical trials or those receiving treatment with the approval of these treatments in the United States, Asia and Europe.

As for other biologic agents, risk of serious infections and opportunistic infections has been reported, mostly among patients participating in clinical trials^8–14^. As compared with patients using biologics (anti-TNF, abatacept, rituximab and tocilizumab), among those receiving tofacitinib, the rate of herpes zoster infection doubled in a real-world American study^15^. Apart from infection risk, studies to evaluate the risk of serious heart-related events and cancer were planned at the time tofacitinib was approved. Recent concerns about ruxolitinib involved occurrence of non-melanoma skin-cancer and second malignancies^16^ and concerns about tofacitinib and baricitinib involved embolism and thrombotic events^17–22^, intestinal perforations^10,23–26^ and malignancies^13,25,27^. Thus, the EMA Committee for Medicinal Products for Human Use and the FDA added thrombosis to the bariticinib and tofacitinib warnings and precautions^28^ as well as intestinal perforations^29^. Post-marketing reporting constitutes an important source to identify safety signals. In this study, we assessed the safety of the first three approved JAK inhibitors—ruxolitinib, tofacitinib and baricitinib—by using the World Health Organization (WHO) international pharmacovigilance database, VigiBase, which contains more than 24 million individual case safety reports (ICSRs) and classifies adverse events according to the Medical Dictionary for Regulatory Activities (MedDRA). To identify safety concern, we used disproportionality analysis.

## Results

Among the 24,416,850 ICSRs in VigiBase, the number involving JAK inhibitors was 126,815. Tofacitinib had the highest number of reports (supplementary Table S1). Physicians reported 12% to 29% of the ICSRs for JAK inhibitors. In 16.3% of the ICSRs for ruxolitinib, 9.6% for tofacitinib and 12.9% for baricitinib, the adverse events caused or prolonged hospitalization. In 14.0% of the ICSRs for ruxolitinib, 1.9% for tofacitinib and 1.4% for baricitinib, the adverse events caused death. The median number of Preferred Terms (PTs) declared by ICSRs was 2.0 (IQR 1.0–3.0). For patients, the median age was 70, 61 and 61 years for ruxolitinib, tofacitinib and baricitinib reports, respectively. More than 75% of the ICSRs for tofacitinib and baricitinib involved women. Rheumatoid arthritis was most frequently reported in tofacitinib and baricitinib ICSRs (55% and 79.7%, respectively), whereas myelofibrosis and polycythemia vera were reported in ruxolitinib ICSRs (43.5% and 19.3%).

### Adverse events

**Figure 1:**
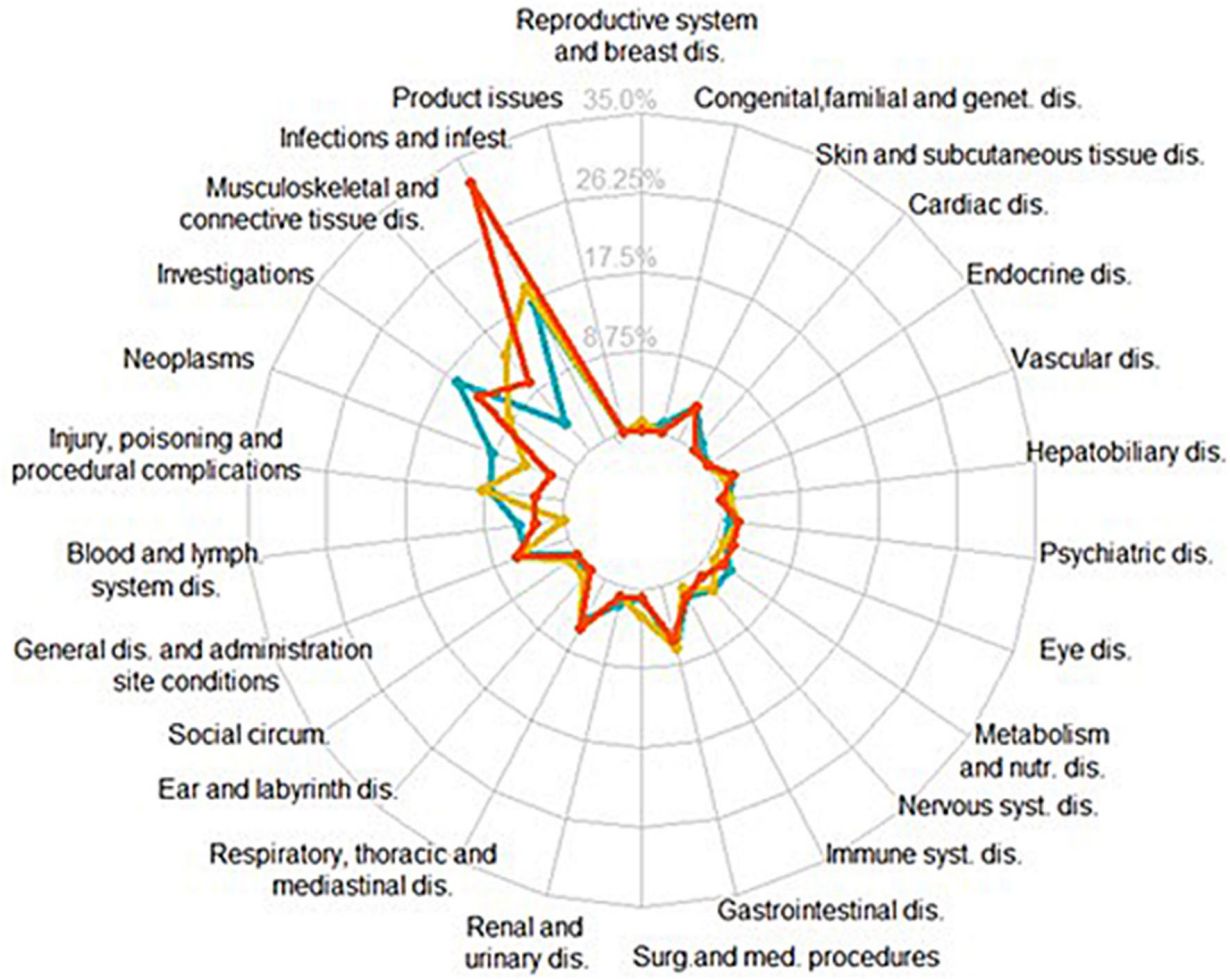
Proportion of Preferred Terms (MedDRA) with positive IC025 related to each JAK inhibitor according to the 27 different System Organ Classes. “Pregancy, puerperium and perinatal conditions” is not represented here because of no positive IC025 observed for any of the three drugs. Yellow curve represents tofacitinib, orange baricitinib and blue ruxolitinib. MedDRA: Medical Dictionary for Regulatory Activities; IC_025_: lower limit of the 95% credibility interval of the information component. A positive IC_025_ is the statistical threshold used in VigiBase.

A total of 376,487 adverse events were reported in the 126,815 ICSRs (including 6179 different PTs). We identified four main System Organ Classes (SOCs) for which adverse event reporting was significantly increased for JAK inhibitors compared with the full database (Fig. 1 and supplementary Table S2): “infections and infestations” (IC025 1.7, i.e. lower limit of the 95% credibility interval of the information component estimated thanks to disproportionality analysis), “musculoskeletal and connective tissue disorders” (IC025 1.1), “investigations” (IC025 0.9), and “neoplasms benign, malignant and unspecified” (IC025 0.8). Six other SOCs (including blood and lymphatic system and respiratory, thoracic and mediastinal disorders) represented also significant increased reporting of adverse event associated with JAK inhibitors (Fig. 1 and supplementary Table S2). We did not find any association for 17 of the 27 different SOCs, including nervous system, psychiatric, vascular, cardiac, skin and subcutaneous tissue disorders (Fig. 1 and supplementary Table S2).

We further described the results regarding (1) infections and infestations, (2) musculoskeletal and connective tissue disorders and (3) neoplasms. We did not describe “investigations” SOC which includes blood test abnormalities because we focused on clinical events rather than isolated biological data. We finally focused on PTs of interest for embolism and thrombosis, gastrointestinal perforations and serious heart-related events.

### Infections and infestations (Table 1, Fig. 2 and supplementary Tables S3 and S4)

**Table 1:**
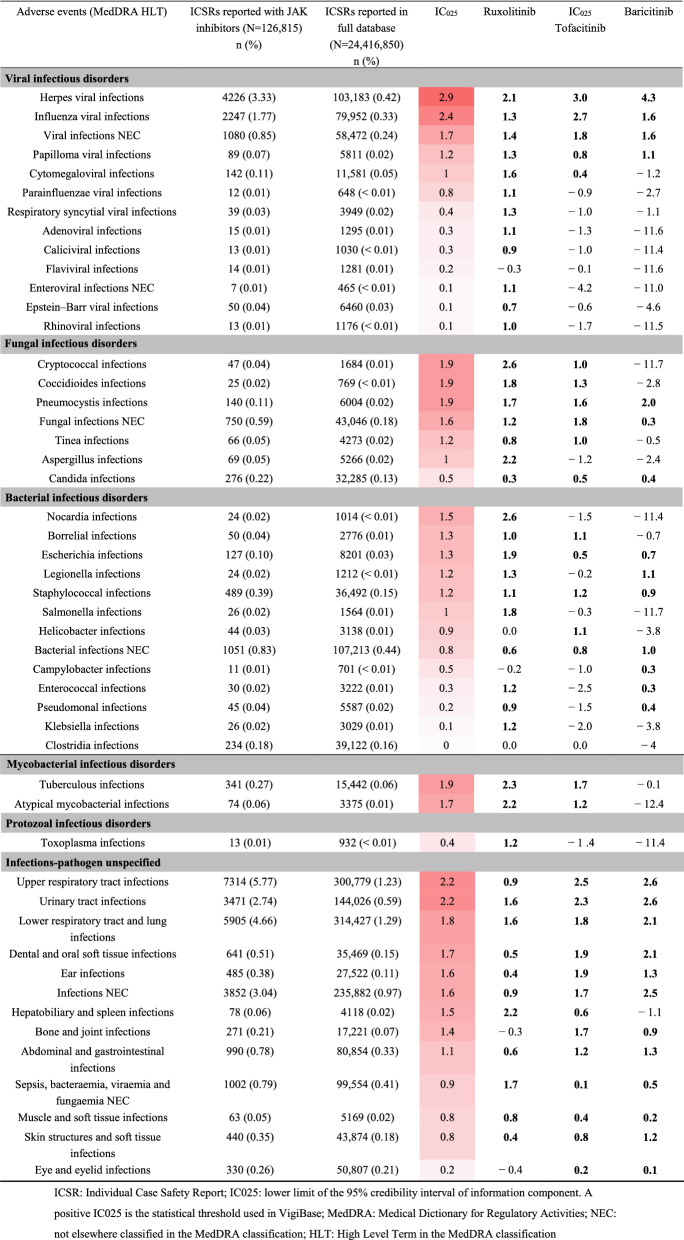
Infectious adverse events related to Janus kinase (JAK) inhibitors.

Significant values are given in bold.

**Figure 2:**
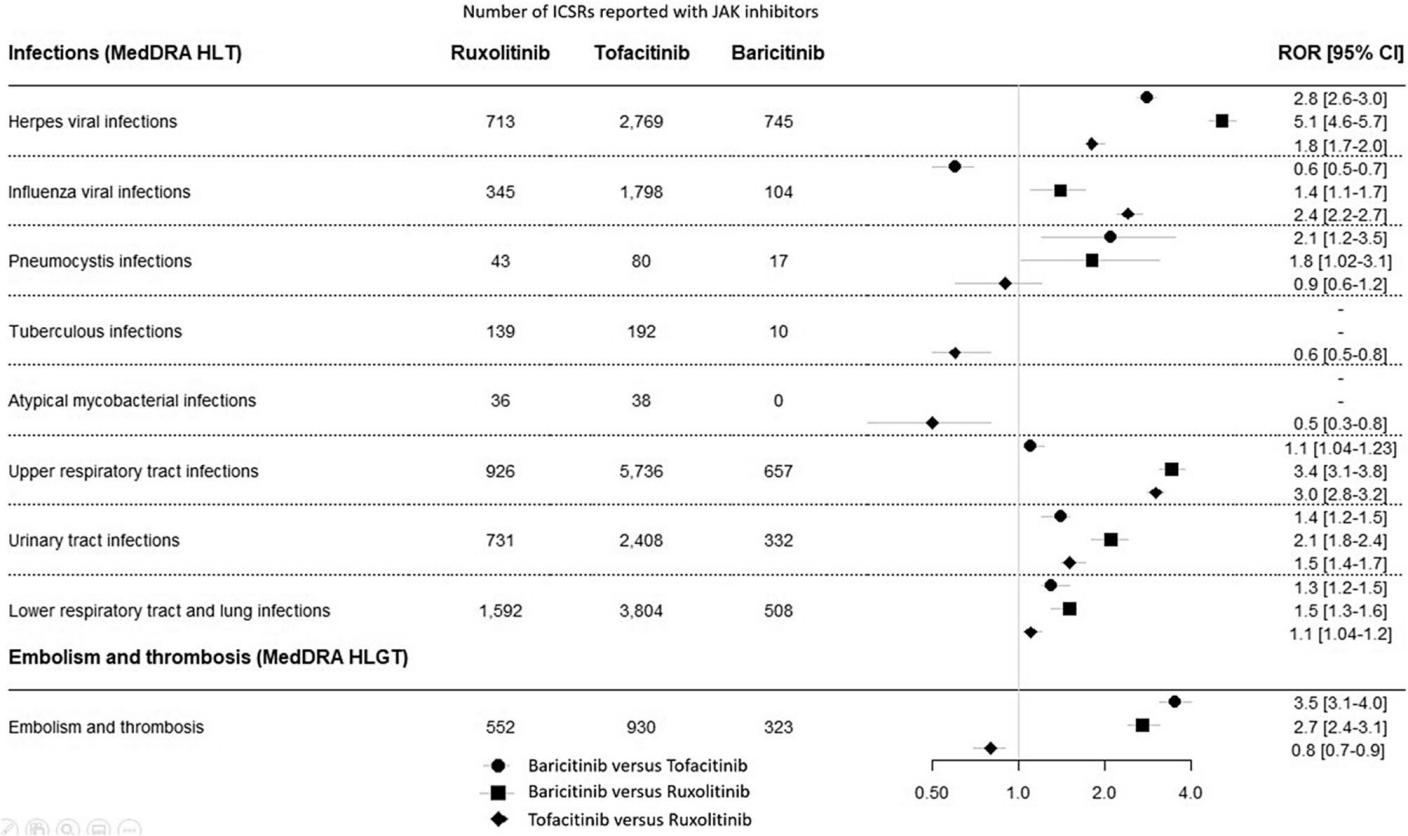
Comparison between the three JAK inhibitors for selected adverse events. *ICSR* Individual Case Safety Report; *ROR [95% CI]* reporting odds ratio and 95% confidence interval. *MedDRA* Medical Dictionary for Regulatory Activities; *HLT* High Level Term in the MedDRA classification; *HLGT* High Level Group Term in the MedDRA classification; *PT* Preferred Term in the MedDRA classification.

The main significant increased reporting of adverse events for viral infections were herpes infections, including herpes viral infections (IC025 2.9), and influenza viral infections (IC025 2.4) (Table 1). We found a differential reporting between the three JAK inhibitors (Fig. 2). Over-reported herpes viral infections were ranked from the highest for baricitinib, then tofacitinib, then ruxolitinib, whereas over-reported influenza viral infections were ranked from the highest for tofacitinib, then baricitinib, then ruxolitinib. High dose of baricitinib was significantly associated with increased reporting of herpes viral infections and influenza viral infections compared with low dose (supplementary Tables S3 and S4). Regarding fungal infectious disorders, we identified pneumocystis infections and cryptococcal and coccidioides infections as significant higher reporting (IC025 1.9 for all three). We found a significant increased reporting concerning pneumocystis infections for each JAK inhibitor, with an over-reporting for baricitinib versus tofacitinib and ruxolitinib (Fig. 2). Similarly, tuberculous and atypical mycobacterial infections had IC025 values close to 2 (1.9 and 1.7, respectively). Tuberculous infections were over-reported for ruxolitinib versus tofacitinib, with no signal observed for baricitinib. Finally, we observed a significant increased reporting of infections according to organ localization: upper respiratory tract infections (IC025 1.9), urinary tract infections (IC025 1.9), and lower respiratory tract and lung infections (IC025 1.9). Over-reported respiratory and urinary tract infections were ranked from the highest for baricitinib, then tofacitinib, then ruxolitinib. High dose of baricitinib was significantly associated with increased reporting of upper respiratory tract infections compared with low dose whereas high dose of tofacitinib was significantly associated with decreased reporting of lower respiratory tract and lung infections and urinary tract infections compared with low dose. No other differences in an over reporting of infections were associated to the dose of either baricitinib or tofacitinib (supplementary Tables S3 and S4).

### Musculoskeletal and connective tissue disorders (supplementary Table S5)

The adverse events “synovial and bursal disorders”, “musculoskeletal and connective tissue deformities” and “joint disorders” were the main significant adverse events reported (IC025 3.4, 2.1 and 1.9, respectively).

### Neoplasms (Table 2 and supplementary Tables S4 and S6)

**Table 2:**
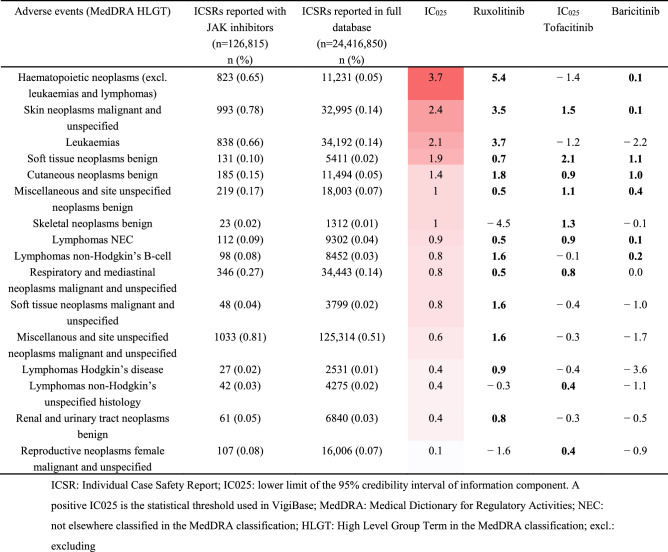
Neoplasm adverse events related to JAK inhibitors.

Significant values are given in bold.

We identified malignant neoplasms for which adverse event reporting was significanty increased: “hematopoietic neoplasms (excluding leukaemias and lymphomas)” (IC_025_ 3.7), “skin neoplasms malignant and unspecified” (IC_025_ 2.4), “leukaemias” (IC_025_ 2.1) and “soft tissue neoplasms benign” (IC_025_ 1.9). “Respiratory and mediastinal neoplasms malignant” also presented a significant increase in reports (IC_025_ 0.8). No differences in an over reporting of neoplams were associated to the dose of either baricitinib or tofacitinib (Supplementary Tables S4 and S6).

### Embolism and thrombosis (Table 3 and Fig. 2 and supplementary Tables S4 and S7)

**Table 3:**
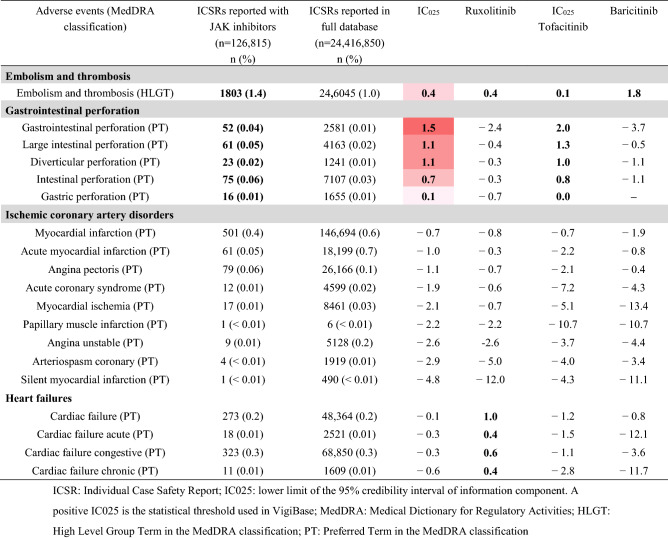
Embolism and thromboembolic, gastrointestinal perforation and major cardiovascular adverse events related to JAK inhibitors.

Significant values are given in bold.

Among the 126,815 ICSRs, 1803 (1.4%) described an embolism and thrombosis adverse event (IC_025_ 0.4). Over-reported embolism and thrombosis adverse events were ranked from the highest for baricitinib, then ruxolitinib, then tofacitinib (Fig. 2). No differences in an over reporting of embolism and thrombosis events were associated to the dose of either baricitinib or tofacitinib (supplementary Tables S4 and S7).

### Gastrointestinal perforation (Table 3 and Fig. 2 and supplementary Table S7)

The JAK inhibitors were associated with higher reporting of “gastrointestinal perforation”, “large intestinal perforation”, “diverticular perforation”, “intestinal perforation” and “gastric perforation”. At the drug level, only tofacitinib had a significant increase of adverse event reporting. Of note, no increase for baricitinib and ruxolitinib did not mean no event: from 3 to 16 events were described for ruxolitinib and from 1 to 4 events for baricitinib.

### Major cardiovascular events (Table 3 and supplementary Table S7)

No major cardiovascular adverse event were associated with higher reporting for JAK inhibitors. Similarly, no cerebrovascular events were reported with JAK inhibitors. At the drug level, only ruxolitinib had a significant increase of reporting for adverse events “cardiac failure”, “cardiac failure acute”, “cardiac failure congestive” and “cardiac failure chronic” compared with the full database.

## Discussion

In this pharmacovigilance study, JAK inhibitors were most commonly associated with infectious adverse events, embolism and thrombosis, neoplasms and gastrointestinal perforation events. We also identified significant increase in adverse event reporting regarding musculoskeletal and connective tissue disorders. Finally, we found no association with major cardiovascular events.

In our study, infections were frequently reported for JAK inhibitors, as was expected according to safety data from clinical trials^24,30^. We found a significant increase in reporting compared with the full database for some microorganisms (viral [herpes and influenza], fungal, and mycobacterial infectious disorders) and two main organ locations (respiratory and urinary tract infections).

Herpes zoster has been identified as a complication of JAK inhibitors in clinical trials^25,26,31,32^ and in a pharmacovigilance study of adverse events reported from the United States^22^. Of note, in our study, herpes viral infections (MedDRA HLT) also include herpes simplex virus. Herpes zoster induced most of the treatment discontinuation due to infections in some clinical trials^25,26^, but few data are available for herpes simplex infections. We observed over-reported herpes viral infections, the highest level for baricitinib, then tofacitinib, then ruxolitinib. Associated risk factors that can affect herpes zoster and herpes simplex infections for patients receiving JAK inhibitors include age, glucocorticoid exposure^25^, other combined therapy, and underlying immunologic dysregulation. For example, herpes zoster/simplex infections were more frequently reported in a pooled safety data analysis of baricitinib in atopic dermatitis than in rheumatoid arthritis^33^. Atopic dermatitis is known to be associated with herpes simplex infections, with a severe form called eczema herpeticum^34^. In our study, the increased risk of herpes viral infections with baricitinib versus the two other JAK inhibitors could be explained by the underlying disorder because the main indication (80%) was rheumatoid arthritis. The risk associated with ruxolitinib was difficult to assess because most patients with haematopoietic neoplasms could have received prophylactic valaciclovir.

Recent concerns about JAK inhibitors involved embolism and thrombosis^17–22^. Although the initial beneficial effect of ruxolitinib for risk of thrombosis was assessed in patients with polycythemia vera and myelofibrosis^35^, lack of evidence remains for this beneficial association. Regarding tofacitinib, in the meta-analyses including 12,410 tofacitinib-exposed patients from completed studies, the incidence rate of venous thromboembolism events was 0.25 (95% CI 0.19–0.33). In our study, we found significant disproportionality analysis results for embolism and thrombosis with the first three approved JAK inhibitors. Over-reported “embolism and thrombosis” adverse events were ranked the highest for baricitinib, then ruxolitinib, then tofacitinib.

These comparisons must be interpreted with caution. Indeed, we did not consider patient characteristics, risk factors for thromboembolism or dose and duration of treatments. In the meta-analysis of clinical trials of tofacitinib, patients with than without baseline cardiovascular risk factors were more likely to experience thromboembolic events^20^. Risk factors were age ≥ 50 years and with at least one criterion (current smoker, high-density lipoprotein level < 40 mg/dL, history of hypertension, diabetes, myocardial infarction or coronary heart disease). Incidence rates in patients without risk factors were very low and most patients who experienced thromboembolic events also had multiple cardiovascular risk factors at baseline. Similarly, all patients with thromboembolic events in a pooled analysis of clinical trials of baricitinib had multiple risk factors^25^. Therefore, the treatment must be adapted to the individual risk.

Regarding neoplasms, we found increased frequency of neoplasm reports and identified “skin neoplasms malignant and unspecified” as significant. The three JAK inhibitors were associated with increased frequency of “skin neoplasms malignant and unspecified”. This is an important finding because previous cohort studies of patients with rheumatoid arthritis did not find a difference between tofacitinib and biologic disease-modifying anti-rheumatic drugs in risk of non-melanoma skin cancer (adjusted hazard ratio 1.04 [95% CI 0.68–1.61])^36^. Rheumatoid arthritis is associated with increased risk of melanoma and non-melanoma skin cancer regardless of the exposure^37,38^. Thus, the increased frequency of “skin neoplasms malignant and unspecified” for ruxolitinib leads to a discussion of a class effect of the JAK inhibitor. “Respiratory and mediastinal neoplasms malignant” was frequently reported for all three JAK inhibitors. This finding confirmed the recent warning from Pfizer for tofacitinib^39^. Indeed, in this warning, the incidence rate of malignancies excluding non-melanoma skin cancer was 1.13 (95% CI 0.94–1.35), with lung cancer as the leading cancer. As for skin neoplasms, this signal concerned all three JAK inhibitors. Lastly, we found a significant increase in reporting for “leukaemias”, in particular for ruxolitinib, which is probably related to the underlying disease.

Some studies have concluded similar incidence rates of malignancies for patients receiving tofacitinib or baricitinib as for those receiving other drugs^40^ and for non-melanoma skin cancer^41^ or malignancies excluding non-melanoma cancer^13,14,25^. ‘Cancer immunoediting’, the process whereby the human immune system destroys cancer cells within the body, is thought to rely upon a variety of cytokines (for example, IFNγ) and cell types (such as NK cells) that could be affected by JAK inhibition^42^. Decrease NK cells could predispose to develop malignancies among patients treated by JAK inhibitors but this effect remains unclear^30^.

Exposure time within trials is relatively limited, and even if pharmacovigilance studies bring interesting data, longer follow-up is needed to further assess malignancy risk and to compare JAK inhibitors with each other.

In our study, we observed increased frequency of gastrointestinal perforations with the three JAK inhibitors. Few cases of gastrointestinal perforation have been reported for patients participating in clinical trials of baricitinib and tofacitinib or those covered by US Medicare/Marketscan^10,23–26^. These few cases were described only among patients with rheumatoid arthritis. To our knowledge, only one clinical trial of ruxolitinib for myelofibrosis reported such an event causing death in a patient in the placebo group^11,43^. In our study, gastrointestinal perforation was over-reported only with tofacitinib. However, cases were also reported for the other two JAK inhibitors. These adverse events would be more frequent for patients with inflammatory bowel diseases. Treatments other than JAK inhibitors such as non-steroidal anti-inflammatory drugs are associated with increased risk of gastrointestinal perforation, which is important to consider with JAK inhibitors.

Finally, the percentage of fatal cases resulted much higher for ruxolitinib than for other JAK inhibitors. We did not perform a detailed analysis and clinical review of the 7000 fatal cases. However, plausible explanation regarding the percentage for ruxolitinib relies on patient characteristics and indications.

Limitations of this study include under-reporting of events and few verifications of the clinical and laboratory tests or radiological findings leading to the diagnosis of the adverse events. Moreover, spontaneous reporting cannot be used to estimate prevalence or incidence of adverse events among patients exposed to drugs. A lack of case-causality constitutes also a main limit. Indeed, individual case safety reports are not fully reliable regarding causal association, due to lack of other potential causes described than suspected drug, and to missing data about time to onset of the adverse event. In this study, we first analyzed the more general level of the MedDRA hierarchy to retain groups of adverse events we further detailed. With this method, we missed potential signals which could be significant at deeper level of the MedDRA hierarchy. Finally, we did not perform subgroup analyses by duration or patient characteristics, which are not available for all ICSRs, but contribute to the occurrence of adverse events. Despite these limitations, pharmacovigilance analyses enable the detection of safety signals. VigiBase relies on data provided by more than 130 countries and enhances the identification of adverse events. Disproportionality analysis is a suitable method to compare spontaneous notifications of groups of drugs with other drugs while avoiding the effect of the extent of use of the product and nature of the adverse events.

## Methods

### Study design and data sources

In this retrospective observational study, pharmacovigilance data were extracted from VigiBase, the World Health Organization (WHO) database of adverse drug reactions reporting, which is managed by the Uppsala Monitoring Centre (UMC). It contains more than 24 million individual case safety reports (ICSRs) submitted by national pharmacovigilance centers from countries around the world since 1967. Different people can report adverse drug reactions: healthcare professionals, patients, pharmaceutical companies. For each ICSR, characteristics of the patient, general administrative information, drugs and reactions are available. A completeness score is also provided, to add a measure of ICSR quality^44^. The likelihood of a causal association is not the same in all reports. The information provided in this study does not represent the opinion of the WHO.

### Procedures

This study included all ICSRs reported from inception to February 28, 2021, with a suspected drug among the following: tofacitinib, baricitinib and ruxolitinib. Each ICSR contains at least one adverse event, which corresponds to the most specific level of the Medical Dictionary for Regulatory Activities (MedDRA) hierarchy: Lowest Level Term (LLT). Each LLT is linked to one Preferred Term (PT), which are themselves grouped into High Level Terms (HLTs). The MedDRA hierarchy thus describes adverse events according to five levels, from the very specific (LLT) to the very general (System Organ Class [SOC]; details are available in supplementary Fig. S1). Each ICSR contains the onset date, end date, seriousness and fatal outcome of the event. A severe adverse event could be any event causing death, being life-threatening, requiring initial or prolonged hospital stay, or leading to persistent or clinically significant disability, congenital anomaly, birth defect or any other medically important condition.

### Statistical analysis

To identify potential safety concern, we used disproportionality analysis, which compares the proportion of each suspected drug-induced adverse event (at different MedDRA levels) reported for a drug or a group of drugs with that for the same adverse event in the full database or for other drugs. Thus, when a proportion of an adverse event is higher for JAK inhibitors than for other drugs, this adverse event could constitute a safety concern. Two main estimations of the disproportionality analysis can be used: the information component (IC) for comparing to the full database or to other drugs and reporting odds ratios (RORs) for comparing drugs belonging to the same group of drugs. The IC was developed and validated by the UMC; it relies on a Bayesian confidence propagation neural network^45^ and the formula is as follows:

$$IC=log2\frac{{N}_{observed}+0.5}{{N}_{expected}+0.5}$$

in which ${N}_{expected}$ is estimated by ${N}_{expected}=\frac{{N}_{drug}\times {N}_{effect}}{{N}_{total}}$, ${N}_{drug}$ is the total number of reports involving the drug studied, and ${N}_{effect}$ is the total number of reports for the adverse events, regardless of drug.

If the corresponding lower end of the 95% credibility interval (IC_025_) positive^46^, the adverse event could be considered a significant signal. This threshold has been used in the UMC and in different signal detection studies. Disproportionality analysis with the IC is illustrated in supplementary Fig. S1.

Disproportionality analysis relies on the ROR for drugs belonging to the same group. We detail the formula with the JAK inhibitors in supplementary Table S8, with corresponding 95% confidence intervals (95% CIs).

We first estimated the IC_025_ for adverse events related to JAK inhibitors at the more general level of the MedDRA hierarchy (SOC). Then, for the SOC with a positive IC_025_, we detailed the IC_025_ for adverse events at the therapeutic class level (JAK inhibitors) and for each drug at different MedDRA levels: High Level Group Terms (HLGTs) and HLTs. Finally, we focused on warnings by regulatory agencies: infections, embolism and thrombosis, serious heart-related events, gastrointestinal perforations. For selected adverse events with a positive IC_025_ for the three JAK inhibitors, we calculated RORs and 95% CIs. For selected adverse events with a positive IC_025_ for tofacitinib and baricitinib, we also estimated the IC_025_ of infections, embolism and thrombosis, serious heart-related events, gastrointestinal perforations according to their doses: high dose over 2 mg per day and 5 mg per day for baricitinb and tofacitinib, respectively; low-dose either. Lastly, we calculated ROR and 95% CIs for these previous adverse events using low-dose as reference. Quantitative variables are described with median (interquartile range) and categorical variables with number (percentage). Analyses involved using R 3.6.2.

## Conclusion

In this international study, we identified significant increase in reporting of adverse event for the first three marketed JAK inhibitors compared with reporting of adverse eventsfor other drugs.. We confirmed some adverse effects such as infectious events and embolism and thrombosis which were already known and mentioned among cautions for use. Our results also lead to increase vigilance regarding malignancies for ruxolitinib, tofacitinib and baricitinib as well as gastrointestinal perforations for tofacitinib. We found no association with major cardiovascular events. Longer follow-up and observational studies will be helpful to improve knowledge about these risks among patients with other risk factors and treatments.

## Supplementary Information

Supplementary Information.

## Data Availability

All relevant data were included in the manuscript. Data sharing not applicable.
