# Supplementary material for: Adverse events associated with JAK inhibitors in 126,815 reports from the WHO pharmacovigilance database
Source: Sci Rep. 2022 May 3;12:7140. doi: 10.1038/s41598-022-10777-w (PMC9065106; doi:10.1038/s41598-022-10777-w)
Supplement: Supplementary file 1 — Supplementary Information. [file 41598_2022_10777_MOESM1_ESM.pdf]

Adverse events associated with JAK inhibitors in 126,815 reports from the WHO pharmacovigilance database

Léa Hoisnard, Bénédicte Lebrun-Vignes, Sébastien Maury, Matthieu Mahevas, Khalil El Karoui, Lydia Roy, Anissa Zarour, Marc Michel, José L Cohen, Aurélien Amiot, Pascal Claudepierre, Pierre Wolkenstein, Philippe Grimbert, Emilie Sbidian

Supplementary Table S1. Characteristics of ICSRs involving Janus kinase (JAK) inhibitors.

	Ruxolitinib<br>N=37,431<br>n (%)	Tofacitinib<br>N=81,130<br>n (%)	Baricitinib<br>N=8,289<br>n (%)
Completeness score, median (IQR)	0.22 (0.15 ; 0.40)	0.32 (0.22 ; 0.41)	0.38 (0.32 ; 0.63)
Region reporting			
North America	28,204 (75.3)	72,727 (89.6)	938 (11.3)
South America	486 (1.3)	1,803 (2.2)	17 (0.2)
Europe	5,592 (14.9)	4,163 (5.1)	6,826 (82.4)
Asia	2,827 (7.6)	1,964 (2.4)	430 (5.2)
Oceania	305 (0.8)	454 (0.6)	42 (0.5)
Africa	17 (<0.1)	19 (<0.1)	36 (0.4)
Reporter qualification			
Physician	11,020 (29.4)	14,541 (17.9)	1,009 (12.2)
Pharmacist	1,530 (4.1)	3,284 (4.0)	314 (3.8)
Other health professional	4,327 (11.6)	18,175 (22.4)	432 (5.2)
Consumer or non-health professional	18,182 (48.6)	41,628 (51.3)	4,375 (52.8)
Lawyer	18 (<0.1)	18 (<0.1)	-
At least 2 different reporters	1,702 (4.5)	2,502 (3.1)	2,031 (24.5)
Missing data	652 (1.7)	982 (1.2)	128 (1.5)
Seriousness criteria			
Caused or prolonged hospitalization	6,083 (16.3)	7,807 (9.6)	1,067 (12.9)
Life threatening	273 (0.7)	412 (0.5)	107 (1.3)
Death	5,222 (14.0)	1,583 (1.9)	119 (1.4)
Disabling or incapacitating	55 (<0.1)	247 (0.3)	26 (0.3)
Congenital anomaly or birth defect	1 (<0.1)	10 (<0.1)	3 (<0.1)
Other medically important condition	6,814 (18.2)	14,702 (18.1)	682 (8.2)
Missing data	18,983 (50.7)	56,369 (69.5)	6,285 (75.8)
Other drug reported	21,914 (58.5)	31,644 (39.0)	3,632 (43.8)
Concomitant	18,823 (50.3)	21,905 (27.0)	3,066 (37.0)
Suspect	3,044 (8.1)	9,550 (11.8)	545 (6.6)
Interaction	47 (0.1)	189 (0.2)	21 (0.3)
Action taken for dealing with adverse event			
Drug withdraw	7,141 (19.1)	24,386 (30.1)	3,677 (44.4)
Dose reduced	2,859 (7.6)	897 (1.1)	181 (2.2)
Dose not changed	8,030 (21.5)	15,356 (18.9)	1,618 (19.5)
Dose increased or drug continued	1,209 (3.2)	321 (0.4)	25 (0.3)
Not applicable	3,526 (9.4)	2,901 (3.6)	82 (1.0)
Missing data	14,666 (39.2)	37,269 (45.9)	2,706 (32.6)

Supplementary Table S1 *Cont.*

	Ruxolitinib<br>N=37,431<br>n (%)	Tofacitinib<br>N=81,130<br>n (%)	Baricitinib<br>N=8,289<br>n (%)
Patient characteristics			
Age, median (IQR)	70 (61.0; 77.0)	61 (53.0; 69.0)	61 (53.0; 70.0)
Missing data	24,085 (64.3)	11,728 (14.5)	3,772 (45.5)
Sex			
Female	9,395 (25.1)	63,245 (78.0)	6,632 (80.0)
Male	10,468 (28.0)	15,114 (18.6)	1,404 (16.9)
Missing data	17,568 (46.9)	2,771 (3.4)	253 (3.1)
Dose			
2mg			1,428 (17.2)
4 mg			4,754 (57.4)
5mg	7,142 (19.1)	31,497 (38.8)	
10 mg	10,723 (28.7)	36,116 (44.5)	
15 mg	5,190 (13.9)		
20 mg	6,740 (18.0)		
Other doses	2,880 (7.7)	787 (1.0)	35 (0.4)
Missing data	4,756 (12.6)	12,730 (15.7)	2,072 (25.0)
Indications			
Rheumatoid arthritis	3 (<0.1)	44,607 (55.0)	6,605 (79.7)
Psoriatic arthritis		2,079 (2.6)	31 (0.4)
Crohn, ulcerative colitis	2 (<0.1)	2,160 (2.7)	0
Other auto-immune and/or auto-inflammatory diseases	25 (<0.1)	1,684 (2.1)	100 (1.2)
Oncology	1,373 (3.7)	37 (<0.1)	4 (<0.1)
Myelofibrosis	16,272 (43.5)	0	0
Polycythemia vera	7,216 (19.3)	0	0
Essential thrombocythemia	819 (2.2)	0	0
Splenomegaly	98 (0.3)	0	0
Graft versus Host Disease	720 (2.0)	0	0
Other indications	5,843 (15.6)	3,961 (4.9)	1,375 (16.6)
Missing data	5,060 (13.5)	26,602 (32.8)	174 (2.1)

Supplementary Table S2. All adverse events related to JAK inhibitors described at the System Organ Class (SOC) level.

Adverse events (MedDRA SOC)	ICSRs reported with JAK inhibitors (N=126,815) n (%)	ICSRs reported in full database (N=24,416,850) n (%)	IC_{025}
**Infections and infestations**	30,317 (23.9)	1,755,936 (7.2)	1.7
**Musculoskeletal and connective tissue disorders**	21,848 (17.2)	1,880,833 (7.7)	1.1
**Investigations**	21,458 (16.9)	2,201,319 (9.0)	0.9
**Neoplasms benign, malignant and unspecified**	5,267 (4.2)	547,615 (2.2)	0.8
**Injury, poisoning and procedural complications**	24,305 (19.2)	2,755,341 (11.3)	0.7
**Blood and lymphatic system disorders**	8,036 (6.3)	93,8142 (3.8)	0.7
**General disorders and administration site conditions**	55,011 (43.4)	7,521,684 (30.8)	0.5
**Social circumstances**	1,242 (0.9)	164,817 (0.7)	0.5
**Ear and labyrinth disorders**	1,870 (1.5)	271,645 (1.1)	0.3
**Respiratory, thoracic and mediastinal disorders**	13,630 (10.8)	2,038,660 (8.4)	0.3
Renal and urinary disorders	3715 (2.9)	708,591 (2.9)	-0.0
Surgical and medical procedures	2,005 (1.6)	374,614 (1.5)	-0.0
Gastrointestinal disorders	21,275 (16.8)	4,441,451 (18.2)	-0.1
Immune system disorders	3,427 (2.7)	750,946 (3.1)	-0.2
Nervous system disorders	18,489 (14.6)	4,301,631 (17.6)	-0.3
Metabolism and nutrition disorders	4,317 (3.4)	960,979 (3.9)	-0.3
Eye disorders	3,362 (2.7)	842,522 (3.5)	-0.4
Psychiatric disorders	7,227 (5.7)	1,851,657 (7.6)	-0.4
Hepatobiliary disorders	1,559 (1.2)	413,778 (1.7)	-0.5
Vascular disorders	4943 (3.9)	1,284,060 (5.3)	-0.5
Endocrine disorders	358 (0.3)	99,678 (0.4)	-0.7
Cardiac disorders	3,376 (2.7)	1,183,282 (4.9)	-0.9
Skin and subcutaneous tissue disorders	11,847 (9.3)	4,568,576 (18.7)	-1.0
Congenital, familial and genetic disorders	176 (0.1)	78,317 (0.3)	-1.4
Reproductive system and breast disorders	848 (0.7)	495,867 (2.0)	-1.7
Product issues	396 (0.3)	566,972 (2.3)	-3.0
Pregnancy, puerperium and perinatal conditions	44 (0.03)	145,643 (0.6)	-4.5

ICSR: Individual Case Safety Report; IC025: lower limit of the 95% credibility interval of the information component. A positive IC025 is the statistical threshold used in VigiBase; MedDRA: Medical Dictionary for Regulatory Activities; SOC: System Organ Class in the MedDRA classification

Supplementary Table S3. Infectious adverse events related to Janus kinase (JAK) inhibitors, according to dose of the drug.

Adverse events (MedDRA HLT)	JAK inhibitors (IC_{025})	Ruxolitinib (IC_{025})	Tofacitinib (IC_{025})			Baricitinib (IC_{025})		
			Total	Low dose	High dose	Total	Low dose	High dose
Viral infectious disorders								
Herpes viral infections	2.9	**2.1**	**3.0**	**2.9**	**2.7**	**4.3**	**3.7**	**4.3**
Influenza viral infections	2.4	**1.3**	**2.7**	**2.9**	**2.7**	**1.6**	**0.2**	**1.9**
Viral infections NEC	1.7	**1.4**	**1.8**	**2.0**	**1.7**	**1.6**	**0.7**	**1.8**
Papilloma viral infections	1.2	**1.3**	**0.8**	0.7	-0.5	**1.1**	-2.9	**1.0**
Cytomegaloviral infections	1	**1.6**	**0.4**	-1.2*	-0.1*	-1.2	-3.4*	-4.6*
Parainfluenzae viral infections	0.8	**1.1**	-0.9	-1.6	-3.7	-2.7	-10.8	-11.0
Respiratory syncytial viral infections	0.4	**1.3**	-1.0	-14.1	-0.1	-1.1	-11.2	-1.5
Adenoviral infections	0.3	**1.1**	-1.3	-4.3	-1.5	-11.6	-10.9	-11.2
Caliciviral infections	0.3	**0.9**	-1.0	-1.1	-2.2	-11.4	-10.8	-11.1
Flaviviral infections	0.2	-0.3	-0.1	-2.3*	-1.4*	-11.6	-10.9	-11.2
Enteroviral infections NEC	0.1	**1.1**	-4.2	-11.8	-11.9	-11.0	-10.7	-10.9
Epstein-Barr viral infections	0.1	**0.7**	-0.6	-2.2*	-1.4*	-4.6	-11.5	-4.0
Rhinoviral infections	0.1	**1.0**	-1.7	-12.7	-0.7	-11.5	-10.8	-11.2
Fungal infectious disorders								
Cryptococcal infections	1.9	**2.6**	**1.0**	-0.1*	-0.6*	-11.7	-10.9	-11.4
Coccidioides infections	1.9	**1.8**	**1.3**	**1.1**	**0.3**	-2.8	-2.3	-11.0
Pneumocystis infections	1.9	**1.7**	**1.6**	**0.7**	**1.5**	**2.0**	-1.0	**2.0**
Fungal infections NEC	1.6	**1.2**	**1.8**	**1.9**	**1.6**	**0.3**	-1.1*	-0.8*
Tinea infections	1.2	**0.8**	**1.0**	**0.7**	**0.4**	-0.5	-11.2	-0.6
Aspergillus infections	1	**2.2**	-1.2	-1.9	-1.7	-2.4	-11.3*	-1.7*
Candida infections	0.5	**0.3**	**0.5**	**0.5**	**0.4**	**0.4**	**0.1**	**0.2**
Bacterial infectious disorders								
Nocardia infections	1.5	**2.6**	-1.5	-12.5*	-4.1*	-11.4	-10.8	-11.1
Borrelial infections	1.3	**1.0**	**1.1**	**0.5**	**0.4**	-0.7	-11.1	-0.2
Escherichia infections	1.3	**1.9**	**0.5**	**0.1**	**0.2**	**0.7**	-1.2	**0.3**
Legionella infections	1.2	**1.3**	-0.2	-1.2*	-2.4*	1.1	-2.3*	-0.8*
Staphylococcal infections	1.2	**1.1**	**1.2**	**1.3**	**1.0**	**0.9**	-0.4	**1.0**
Salmonella infections	1	**1.8**	-0.3	-0.9	-1.0	-11.7	-10.9	-11.3
Helicobacter infections	0.9	0.0	1.1	1.5	-0.7	-3.8	-11.1	-3.3
Bacterial infections NEC	0.8	**0.6**	**0.8**	**0.9**	**0.7**	**1.0**	**0.4**	**1.2**
Campylobacter infections	0.5	-0.2	-1.0	-3.6*	-3.8*	**0.3**	-10.8*	-2.5*
Enterococcal infections	0.3	**1.2**	-2.5	-3.4*	-14.0*	**0.3**	-2.6*	-1.4*
Pseudomonal infections	0.2	**0.9**	-1.5	-0.6	-14.8	**0.4**	-0.9	-0.2
Klebsiella infections	0.1	**1.2**	-2.0	-1.2	-5.5	-3.8	-11.1	-3.3
Clostridia infections	0	0.0	0.0	-0.5	0.0	-4	-13.1	-3.2

Supplementary Table S3. *Cont.*

Adverse events (MedDRA HLT)	JAK inhibitors (IC_{025})	Ruxolitinib (IC_{025})	Tofacitinib (IC_{025})			Baricitinib (IC_{025})		
			Total	Low dose	High dose	Total	Low dose	High dose
Mycobacterial infectious disorders								
Tuberculous infections	1.9	**2.3**	**1.7**	**1.2**	**1.7**	-0.1	-1.7	**0.1**
Atypical mycobacterial infections	1.7	**2.2**	**1.2**	**0.7**	**0.4**	-12.4	-11.1	-11.9
Protozoal infectious disorders								
Toxoplasma infections	0.4	**1.2**	-1 .4	-12.4*	-2.1*	-11.4	-10.8	-11.1
Infections-pathogen unspecified								
Upper respiratory tract infections	2.2	**0.9**	**2.5**	**2.6**	**2.6**	**2.6**	**1.9**	**2.6**
Urinary tract infections	2.2	**1.6**	**2.3**	**2.5**	**2.0**	**2.6**	**2.2**	**2.6**
Lower respiratory tract and lung infections	1.8	**1.6**	**1.8**	**2.1**	**1.6**	**2.1**	**2.2**	**2.2**
Dental and oral soft tissue infections	1.7	**0.5**	**1.9**	**2.0**	**1.7**	**2.1**	**1.4**	**2.0**
Ear infections	1.6	**0.4**	**1.9**	**1.9**	**1.9**	**1.3**	-0.6	**1.3**
Infections NEC	1.6	**0.9**	**1.7**	**1.8**	**1.6**	**2.5**	**2.3**	**2.7**
Hepatobiliary and spleen infections	1.5	**2.2**	**0.6**	**0.3**	**0.4**	-1.1	-11.2	-1.6
Bone and joint infections	1.4	-0.3	**1.7**	**1.9**	**1.3**	**0.9**	-0.8	**1.1**
Abdominal and gastrointestinal infections	1.1	**0.6**	**1.2**	**1.3**	**1.1**	**1.3**	**0.5**	**1.1**
Sepsis, bacteraemia, viraemia and fungaemia NEC	0.9	**1.7**	**0.1**	**0.3**	-0.2	**0.5**	**0.8**	**0.3**
Muscle and soft tissue infections	0.8	**0.8**	**0.4**	**0.4**	-0.5	**0.2**	-11.3*	-0.1*
Skin structures and soft tissue infections	0.8	**0.4**	**0.8**	**0.8**	0.5	**1.2**	-0.6	**1.5**
Eye and eyelid infections	0.2	-0.4	**0.2**	**0.4**	-0.1	**0.1**	-2.0	**0.1**

*Due to missing value for dose, IC_{025} is probably biaised

IC025: lower limit of the 95% credibility interval of information component. A positive IC025 is the statistical threshold used in VigiBase; MedDRA: Medical Dictionary for Regulatory Activities; high dose: 4mg for baricitinib and 10mg for tofacitinib; low dose: 2mg for baricitinib and 5mg for tofacitinib; NEC: not elsewhere classified in the MedDRA classification; HLT: High Level Term in the MedDRA classification

Supplementary Table S4. Comparison between doses of baricitinib and tofacitinib for selected adverse events.

Adverse events	Baricitinib high dose ROR[95% CI]	Tofacitinib high dose ROR[95% CI]
Infections (MedDRA HLT)		
Herpes viral infections	**1.3 [1.1-1.6]**	0.9 [0.8-1.0]
Influenza viral infections	**2.1 [1.1-4.0]**	0.9 [0.8-1.0]
Pneumocystis infections	1.8 [0.4-8.1]	1.5 [0.9-2.6]
Tuberculosis infections	1.2 [0.3-5.7]	1.3 [1.0-1.8]
Atypical mycobacterial infections		0.8 [0.4-1.7]
Upper respiratory tract infections	**1.4 [1.1-1.8]**	1.0 [0.9-1.1]
Urinary tract infections	1.1 [0.8-1.5]	**0.7 [0.7-0.8]**
Lower respiratory tract and lung infections	0.9 [0.7-1.1]	**0.7 [0.6-0.7]**
Neoplasms (MedDRA HLGT)		
Skin neoplasms malignant and unspecified	4.5 [0.6-34.2]	0.8 [0.7-1.1]
Soft tissue neoplasms benign	1.2 [0.1-10.8]	0.7 [0.5-1.1]
Respiratory and mediastinal neoplasms malignant and unspecified	0.4 [0.2-1.1]	1.0 [0.8-1.4]
Embolism and thrombosis (MedDRA HLGT)		
Embolism and thrombosis	0.9 [0.6-1.2]	1.1 [1.0-1.3]

ROR [95% CI]: reporting odds ratio and 95% confidence interval; MedDRA: Medical Dictionary for Regulatory Activities; HLGT: High Level Group Term in the MedDRA classification; HLT:High Level Term in the MedDRA classification; high dose: 4mg for baricitinib and 10mg for tofacitinib

Supplementary Table S5. Musculoskeletal adverse events related to JAK inhibitors.

Adverse events (MedDRA HLGT)	ICSRs reported with JAK inhibitors (n=126,815) n (%)	ICSRs reported in full database (n=24,416,850) n (%)	IC_{025}	Ruxolitinib	Tofacitinib	Baricitinib
**Synovial and bursal disorders**	855 (0.67)	14,155 (0.06)	3.4	**0.2**	**3.9**	**1.8**
**Musculoskeletal and connective tissue deformities (including intervertebral disc disorders)**	1,249 (0.98)	53,825 (0.22)	2.1	**0.6**	**2.5**	**1.1**
**Joint disorders**	13,092 (10.32)	647,691 (2.65)	1.9	**0.5**	**2.3**	**2.1**
Fractures	2,125 (1.68)	150,506 (0.62)	1.4	**0.8**	**1.6**	**0.4**
Musculoskeletal and connective tissue disorders NEC	12,420 (9.79)	896,830 (3.67)	1.4	**0.9**	**1.6**	**0.6**
Tendon, ligament and cartilage disorders	777 (0.61)	65,001 (0.27)	1.1	0.0	1.4	0.5
Bone disorders (excl. congenital and fractures)	1,999 (1.58)	196,240 (0.80)	0.9	1.1	0.8	0.0
Connective tissue disorders (excl. congenital)	365 (0.29)	40,567 (0.17)	0.6	-0.5	1.0	-1.7
Musculoskeletal and connective tissue neoplasms	120 (0.09)	17,862 (0.07)	0.1	0.7	-0.6	-0.5

ICSR: Individual Case Safety Report; IC025: lower limit of the 95% credibility interval of information component. A positive IC025 is the statistical threshold used in VigiBase; MedDRA: Medical Dictionary for Regulatory Activities; NEC: not elsewhere classified in the MedDRA classification; HLGT: High Level Group Term in the MedDRA classification; excl.: excluding

Supplementary Table S6 . Neoplasm adverse events related to JAK inhibitors, according to dose of the drug.

Adverse events (MedDRA HLGT)	JAK inhibitors (IC_{025})	Ruxolitinib (IC_{025})	Tofacitinib (IC_{025})			Baricitinib (IC_{025})		
			Total	Low dose	High dose	Total	Low dose	High dose
**Haematopoietic neoplasms (excl. leukaemias and lymphomas)**	3.7	**5.4**	-1.4	-4.1	-1.4	**0.1**	**0.8**	-0.9
**Skin neoplasms malignant and unspecified**	2.4	**3.5**	**1.5**	**1.6**	**1.3**	**0.1**	-4.4	**0.3**
**Leukaemias**	2.1	**3.7**	-1.2	-1.2	-2.0	-2.2	-4.5	-2.4
**Soft tissue neoplasms benign**	1.9	**0.7**	**2.1**	**2.2**	**1.8**	**1.1**	-2.9*	-0.2*
Cutaneous neoplasms benign	1.4	**1.8**	**0.9**	**0.9**	**0.3**	**1.0**	-3.4	**0.9**
Miscellaneous and site unspecified neoplasms benign	1	**0.5**	**1.1**	**1.2**	**1.0**	**0.4**	-1.8	**0.2**
Skeletal neoplasms benign	1	-4.5	**1.3**	**0.8**	**1.1**	-0.1	-10.9*	-2.7*
Lymphomas NEC	0.9	**0.5**	**0.9**	**0.8**	-0.1	**0.1**	-3.2	-0.2
Lymphomas non-Hodgkin's B-cell	0.8	**1.6**	-0.1	-0.4	-1.0	**0.2**	-3.1	**0.3**
Respiratory and mediastinal neoplasms malignant and unspecified	0.8	**0.5**	**0.8**	**0.7**	**0.7**	0.0	**0.3**	-0.5
Soft tissue neoplasms malignant and unspecified	0.8	**1.6**	-0.4	-0.8	-0.7	-1.0	-2.7*	-12.0*
Miscellanous and site unspecified neoplasms malignant and unspecified	0.6	**1.6**	-0.3	-0.2	-2.5	-1.7	-2.0*	-2.5*
Lymphomas Hodgkin's disease	0.4	**0.9**	-0.4	-1.4	-0.7	-3.6	-11.0	-3.1
Lymphomas non-Hodgkin's unspecified histology	0.4	-0.3	**0.4**	**0.9**	-1.1	-1.1	-11.2	-1.6
Renal and urinary tract neoplasms benign	0.4	**0.8**	-0.3	-0.5	-0.2	-0.5	-3.0	-1.1
Reproductive neoplasms female malignant and unspecified	0.1	-1.6	**0.4**	**0.3**	-0.1	-0.9	-1.7	-2.0

*Due to missing value for dose, IC_{025} is probably biaised

IC025: lower limit of the 95% credibility interval of information component. A positive IC025 is the statistical threshold used in VigiBase; MedDRA: Medical Dictionary for Regulatory Activities; high dose: 4mg for baricitinib and 10mg for tofacitinib; low dose: 2mg for baricitinib and 5mg for tofacitinib; NEC: not elsewhere classified in the MedDRA classification; HLGT: High Level Group Term in the MedDRA classification; excl.: excluding

Supplementary Table S7. Embolism and thromboembolic, gastrointestinal perforation adverse events related to JAK inhibitors, according to dose of the drug.

Adverse events (MedDRA classification)	JAK inhibitors (IC_{025})	Ruxolitinib (IC_{025})	Tofacitinib (IC_{025})			Baricitinib (IC_{025})		
			Total	Low dose	High dose	Total	Low dose	High dose
Embolism and thrombosis								
**Embolism and thrombosis (HLGT)**	**0.4**	**0.4**	**0.1**	-0.2	0.0	**1.8**	**1.6**	**1.6**
Gastrointestinal perforation								
**Gastrointestinal perforation (PT)**	**1.5**	-2.4	**2.0**	**0.4***	**0.8***	-3.7	-2.5	-11.7
**Large intestinal perforation (PT)**	**1.1**	-0.4	**1.3**	**0.2**	**1.1**	-0.5	-2.7	-1.6
**Diverticular perforation (PT)**	**1.1**	-0.3	**1.0**	-1.3*	**0.4***	-1.1	-10.8*	-2.7*
**Intestinal perforation (PT)**	**0.7**	-0.3	**0.8**	**0.4**	0.0	-1.1	-1.1	-2.1
**Gastric perforation (PT)**	**0.1**	-0.7	0.0	-2.6	**0.1**	-11.7	-10.9	-11.4
Ischemic coronary artery disorders								
Myocardial infarction (PT)	-0.7	-0.8	-0.7	-0.5	-1.1	-1.9	-2.2	-2.3
Acute myocardial infarction (PT)	-1.0	-0.3	-2.2	-2.6	-2.2	-0.8	-3.8	-1.0
Angina pectoris (PT)	-1.1	-0.7	-2.1	-2.2	-2.7	-0.4	-2.2	-0.8
Acute coronary syndrome (PT)	-1.9	-0.6	-7.2	-14.3	-6.0	-4.3	-2.8	-12.1
Myocardial ischemia (PT)	-2.1	-0.7	-5.1	-4.7	-6.8	-13.4	-11.6	-12.8
Papillary muscle infarction (PT)	-2.2	-2.2	-10.7	-10.7	-10.7	-10.7	-10.7	-10.7
Angina unstable (PT)	-2.6	-2.6	-3.7	-14.5	-3.2	-4.4	-11.3	-12.2
Arteriospasm coronary (PT)	-2.9	-5.0	-4.0	-13.2	-4.9	-3.4	-10.9	-11.5
Silent myocardial infarction (PT)	-4.8	-12.0	-4.3	-3.3	-11.9	-11.1	-10.7	-10.9
Heart failures								
Cardiac failure (PT)	-0.1	**1.0**	-1.2	-1.2	-1.7	-0.8	-0.8	-1.4
Cardiac failure acute (PT)	-0.3	**0.4**	-1.5	-5.1	-1.1	-12.1	-11.0	-11.6
Cardiac failure congestive (PT)	-0.3	**0.6**	-1.1	-1.0	-1.3	-3.6	-13.8	-3.3
Cardiac failure chronic (PT)	-0.6	**0.4**	-2.8	-4.5	-2.7	-11.7	-10.9	-11.4

*Due to missing value for dose, IC_{025} is probably biaised

IC025: lower limit of the 95% credibility interval of information component. A positive IC025 is the statistical threshold used in VigiBase; MedDRA: Medical Dictionary for Regulatory Activities; high dose: 4mg for baricitinib and 10mg for tofacitinib; low dose: 2mg for baricitinib and 5mg for tofacitinib; HLGT: High Level Group Term in the MedDRA classification; PT: Preferred Term in the MedDRA classification

Supplementary Table S8. Example of calculation of the reporting odds ratios (RORs) from VigiBase.

	ICSRs with the suspected adverse events	ICSRs without the suspected adverse events
ICSRs with suspected drug	A	B
ICSRs with comparator drug	C	D

A: Number of Individual Case Safety Reports (ICSRs) of drug-related adverse events of interest (e.g., embolism and thrombosis) associated with a suspected drug (e.g., one of the three JAK inhibitors, baricitinib)

B: Number of ICSRs of other adverse events associated with a suspected drug (e.g., one of the three JAK inhibitors, baricitinib)

C: Number of ICSRs associated with a comparator drug (e.g., another JAK inhibitor, ruxolitinib)

D: Number of ICSRs of other adverse events associated with a comparator drug (e.g., another JAK inhibitor, ruxolitinib)

$$\text{ROR}=\frac{\frac{A}{C}}{\frac{B}{D}}=\frac{AD}{BC}$$

Supplementary Figure S1. Medical Dictionary for Regulatory Activities (MedDRA) hierarchy and disproportionality analysis of adverse events with information component (IC).

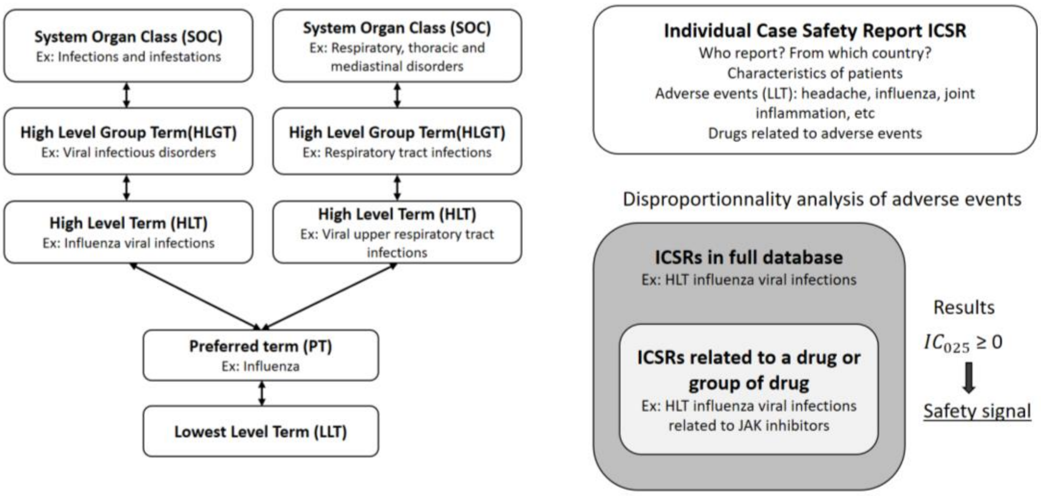

One preferred term could belong to one or more High Level Term (HLT), High Level Group Term (HLGT) or System Organ Class (SOC). ICSR: Individual Case Safety Report; IC_{025} is the lower limit of the 95% credibility interval of the information component. A positive IC_{025} is the statistical threshold used in VigiBase. PT: preferred term.
